# Projections of SDEs onto submanifolds

**DOI:** 10.1007/s41884-022-00093-7

**Published:** 2023-02-02

**Authors:** John Armstrong, Damiano Brigo, Emilio Ferrucci

**Affiliations:** 1 Department of Mathematics, King’s College London, London, UK; 2 Department of Mathematics, Imperial College London, London, UK; 3 Mathematical Institute, University of Oxford, Oxford, UK

**Keywords:** Ito, Stratonovich, Projection

## Abstract

In Armstrong et al. (Proc Lond Math Soc (3) 119(1):176–213, 2019) the authors define three projections of ${\mathbb {R}}^d$-valued stochastic differential equations (SDEs) onto submanifolds: the Stratonovich, Itô-vector and Itô-jet projections. In this paper, after a brief survey of SDEs on manifolds, we begin by giving these projections a natural, coordinate-free description, each in terms of a specific representation of manifold-valued SDEs. We proceed by deriving formulae for the three projections in ambient ${\mathbb {R}}^d$-coordinates. We use these to show that the Itô-vector and Itô-jet projections satisfy respectively a weak and mean-square optimality criterion “for small t”: this is achieved by solving constrained optimisation problems. These results confirm, but do not rely on the approach taken in Armstrong et al. (Proc Lond Math Soc (3) 119(1):176–213, 2019), which is formulated in terms of weak and strong Itô–Taylor expansions. In the final section we exhibit examples showing how the three projections can differ, and explore alternative notions of optimality.

## Introduction

Consider the following problem: we are given an autonomous ODE

1 $$\begin{aligned} \dot{X}_t = F(X_t) \end{aligned}$$

in ${\mathbb {R}}^d$, and a smooth embedded manifold $M \hookrightarrow {\mathbb {R}}^d$. Let $\pi$ be the metric projection of a tubular neighbourhood of *M* onto *M* (see (41) below). We seek an *M*-valued ODE, i.e. a vector field ${\overline{F}}$ on *M*, tangent at each point to *M*, with the property that the solution to

2 $$\begin{aligned} \dot{Y}_t = {\overline{F}}(Y_t) \end{aligned}$$

is optimal in the sense that the first coefficient of the Taylor expansion in $t = 0$ of either

3 $$\begin{aligned} |Y_t - X_t|^2 \quad \text {or} \quad |Y_t - \pi (X_t)|^2 \end{aligned}$$

is minimised for any initial condition $X_0 = Y_0 = y_0 \in M$. This requirement represents the slowest possible divergence of *Y* from the original solution *X* (resp. from its metric projection on *M*), subject to the constraint of *Y* arising as the solution of a closed form ODE on *M*. It is an easy exercise (using (44) below) to check that these optimisation problems both result in the same solution, which consists in ${\overline{F}}(y)$ being the orthogonal projection of the vector *F*(*y*) onto the tangent space $T_yM$.

In the stochastic setting, the optimality criteria (3) do not carry over in a straightforward fashion, and in [2] are formulated through the machinery of weak and strong Itô-Taylor expansions, i.e. approximations of solutions to SDEs that use iterated Itô integrals [12]. The idea of projecting an SDE onto a finite dimensional manifold is motivated by the projection method for approximating the solution to non-linear filtering problems described in [4], and [2] gives an example of how one can obtain optimal Gaussian approximations to non-linear filterings by considering the *Itô-jet projection* defined therein. More generally, whenever one considers a perturbation of an SDE whose solutions are known to be confined to a manifold, it would be natural to approximate the solutions to the perturbed problem by projecting onto that manifold. Projection is also likely to be of interest in problems where the geometry of the situation or conservation laws introduce a natural manifold structure.

In this paper we tackle the core problem of [2] through a different perspective, which we proceed to describe. In Sect. 2 we begin with a survey of SDEs on manifolds, and describe three equivalent but distinct ways of writing SDEs on smooth manifolds. In Sect. 3 we prepare the framework for manifolds *M* embedded in ${\mathbb {R}}^d$, and use this framework to study the equations introduced in the previous section, in extrinsic coordinates. In Sect. 4 we associate to each manifold-valued SDE representation a natural projection, which gives rise to an SDE on a submanifold. These projections coincide with the ones introduced in [2], but are described in ambient coordinates instead of local ones: this removes dependency on the chart in the expression for the projected coefficients, which in turn makes it easier to interpret these geometrically, namely to consider their components orthogonal and tangent to the manifold (see (90) below for the expressions of the tangential part of the Itô drift terms). In Sect. 5 we formulate the optimality criteria satisfied by the projections introduced prior, using a weak/mean-square formulation, i.e. by directly minimising the classical Taylor coefficients of the quantities (94), instead of invoking Itô-Taylor expansions as done in [2]: this has the advantage of representing a more tangible property of the solution. Our main theorems 1 and 2 replicate the findings [2, Theorem 4.4 and Theorem 4.7] in this new setting. The fact that the Stratonovich projection does not satisfy either of these optimality criteria is a confirmation of the fact that Itô calculus on manifolds can be of great interest.

In this paper, we tackle the problem of finding the coefficients for the SDE defined intrinsically on manifold, such that the solution best approximates the solution to a given SDE in a certain mean-square or weak sense, for small time. While the formulation and first solution to this problem are original to [2], the independent approach given here has three major advantages: formulae are more easily interpretable and comparable thanks to the use of ambient coordinates, the proof of optimality is streamlined by bypassing the use of stochastic Taylor expansions, and the optimal solutions are shown to coincide with natural notions of projections of intrinsic SDEs on manifolds referenced in the literature, giving them a more solid theoretical underpinning.

## SDEs on manifolds

Let *M* be a smooth manifold; we write *TM* for its tangent bundle and $\Gamma TM$ for its $C^\infty M$-module of sections, i.e. tangent vector fields. A time-homogeneous ODE on *M* consists of a vector field $F \in \Gamma TM$, i.e. *X* is a solution with initial condition $x_0$ if $X_0 = x_0 \text { and } \dot{X}_t = F(X_t)$, where $\dot{X}_t \in T_{X_t}M$ is the tangent vector of the smooth curve *X* at time *t*. This can be written in local coordinates as $\dot{X}^k_t = F^k(X_t)$.

Not too much needs to be changed in order to describe Stratonovich equations. As for the familiar ${\mathbb {R}}^d$-valued case we will also need a driving semimartingale, which, following [6] we take to be valued in another manifold *N*, of dimension *n*. Given a stochastic setup $(\Omega , {\mathcal {F}}_\cdot , P)$ satisfying the usual conditions, a continuous adapted stochastic process $Z :\Omega \times {\mathbb {R}}_{\ge 0} \rightarrow N$ is said to be a *semimartingale* if, for all $f \in C^\infty N$, *f*(*Z*) is a semimartingale. Just as for the ODE case, what is needed to define a Stratonovich SDE in *M* driven by *Z* is a section of some vector bundle: in this case, however, the bundle is no longer just *TM*, but $\text {Hom}(TN,TM) \rightarrow M \times N$, i.e. the vector bundle of linear maps from *TN* to *TM*. An element $F \in \Gamma \text {Hom}(TN,TM)$ corresponds to a smooth map $M \times N \ni (x,z) \mapsto F(x,z) \in \text {Hom}(T_zN, T_xM)$. The Stratonovich SDE

4 $$\begin{aligned} \text {d}X_t = F(X_t,Z_t) \circ \text {d}Z_t \end{aligned}$$

in local coordinates (this requires choosing a chart both on *N* and on *M*) as $\text {d}X_t^k = F^k_\gamma (X_t, Z_t) \circ \text {d}Z^\gamma _t$ on random intervals that make both sides of the expression well defined. We will always use Greek letters as indices for the driving process, and Latin letters as indices for the solution. The key property that allows one to prove that the coordinate formulation of Stratonovich SDEs holds for all other charts (on the intersection of their respective domains) is that Stratonovich equations satisfy the first order chain rule: clearly (4) would not be similarly well defined with Itô integration.

### Example 1

(Stratonovich diffusion) An important example is the case where $N = {\mathbb {R}}_{\ge 0} \times {\mathbb {R}}^n$ and $Z_t = (t,W_t)$, *W* an *n*-dimensional Brownian motion, and *F* not depending explicitly on *W*. This means (4) becomes

5 $$\begin{aligned} \text {d}X_t = \sigma _\gamma (X_t, t) \circ \text {d}W^\gamma _t + b(X_t,t) \text {d}t \end{aligned}$$

for $\sigma _\gamma , b \in \Gamma \text {Hom}(T{\mathbb {R}}_{\ge 0},TM) = C^\infty ({\mathbb {R}}_{\ge 0}, \Gamma TM)$, $\gamma = 1,\ldots , n$. Stratonovich diffusions are sections of the vector bundle

6 $$\begin{aligned} {\begin{matrix}{\text{ Diff }}^{\, n}_{\text{ Strat }} &{}{:}{=} \left\{ F \in {\text{ Hom }}(T({\mathbb {R}}_{\ge 0} \oplus {\mathbb {R}}^n), TM) : \right. \\ &{}\left. \qquad \forall w_1,w_2 \in {\mathbb {R}}^n \ F(t,w_1;x) = F(t,w_2;x) \right\} \rightarrow M \times {\mathbb {R}}_{\ge 0} \end{matrix}} \end{aligned}$$

i.e. elements of the vector space $\Gamma \text {Diff}^{\, n}_\text {Strat}$. Notice that the base space is not $M \times ({\mathbb {R}}_{\ge 0} \times {\mathbb {R}}^n)$, since independence of the Brownian motion allows us to forget the ${\mathbb {R}}^n$ component.

We note that no additional structure on *N* and *M*, apart from their smooth atlas, is needed to define Stratonovich equations. Stratonovich SDEs are the most used in stochastic differential geometry, as they behave well w.r.t. notions of first order calculus: for instance, if there exists an embedded submanifold $M'$ of *M* such that *F*(*y*, *z*) maps to $T_y M'$ for all $z \in N$ and all $y \in M'$, then the solution to the Stratonovich SDE defined by *F* started on $M'$ will remain on $M'$ for the duration of its lifetime.

We now pass to Itô theory on manifolds, as developed in [6, Ch.VI]. The difficulty lies in the second order chain rule of the Itô integral. For this reason, we need to invoke structures of order higher than 1. Let the *second order tangent bundle* of *M*, ${\mathbb {T}}M$, denote the bundle of second order differential operators without a constant term, i.e. given a local chart $\varphi$ containing *x* in its domain, an element of $L_x \in {\mathbb {T}}_x M$ consists of a map

7 $$\begin{aligned} L_x :C^\infty M \rightarrow {\mathbb {R}}, \quad L_x f = L^k_x \frac{\partial f}{\partial \varphi ^k} + L^{ij}_x \frac{\partial ^2 f}{\partial \varphi ^i \partial \varphi ^j} \end{aligned}$$

The coefficients $L^k_x$, $L^{ij}_x$ obviously depend on $\varphi$, but their existence does not; moreover, requiring $L^{ij}_x = L^{ji}_x$ ensures their uniqueness for the given chart $\varphi$. Note that if the $L_x^{ij}$’s vanish $L_x \in T_xM$. ${\mathbb {T}}M$ is given the unique topology and smooth structure that makes the projection ${\mathbb {T}}M \rightarrow M$, $L_x \mapsto x$ a locally trivial surjective submersion. Just as for the first order case, there is an obvious notion of induced bundle map ${\mathbb {T}}f :{\mathbb {T}}N \rightarrow {\mathbb {T}}M$ for $f \in C^\infty (N,M)$. A chart $\varphi$ containing *x* in its domain defines the basis of ${\mathbb {T}}_xM$

8 $$\begin{aligned} \{\partial _x \varphi _k, \partial ^2_x \varphi _{ij} = \partial ^2_x \varphi _{ji} \mid k,i,j = 1,\ldots , n\} \end{aligned}$$

so the dimension of ${\mathbb {T}}M$ (as a vector bundle) is $m + m(m+1)/2$. The fundamental properties of ${\mathbb {T}}M$ are summarised the short exact sequence of vector bundles over *M*
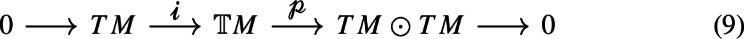
with the third term denoting symmetric tensor product, the first map the obvious inclusion and the second map given by

10 $$\begin{aligned} L_x \mapsto \left( f,g \mapsto \frac{1}{2} (L_x(fg) - f(x)L_xg - g(x)L_xf)\right) \end{aligned}$$

Roughly speaking, this means that ${\mathbb {T}}M$ is “noncanonically the direct sum of *TM* and $TM \odot TM$”. We now wish to define an Itô-type equation using second order tangent bundles instead of ordinary tangent bundles. For this we need a notion of field of maps ${\mathbb {F}}(x,z) :{\mathbb {T}}_z N \rightarrow {\mathbb {T}}_x M$. Since the bundles in question are linear, it is tempting to allow ${\mathbb {F}}(x,z)$ to be an arbitrary linear map, but a more stringent condition is necessary to guarantee well-posedness: the correct requirement is that ${\mathbb {F}}(x,z)$ define a morphism of short exact sequences, i.e. a commutative diagram 
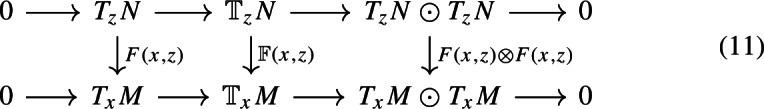
 with $F(x,z) = {\mathbb {F}}(x,z)|_{T_zN}$. ${\mathbb {F}}(x,z)$ is then called a *Schwartz morphism*, and we can then view ${\mathbb {F}}$ as being the section of a sub-fibre bundle $\text {Sch}(N,M)$ of $\text {Hom}({\mathbb {T}}N, {\mathbb {T}}M)$ over $M \times N$ consisting of such maps, which we call the *Schwartz bundle*. Note that $\text {Sch}(N,M)$ is not closed under sum and scalar multiplication taken in the vector bundle $\text {Hom}({\mathbb {T}}N, {\mathbb {T}}M)$, and thus can only be treated as a fibre bundle. Now, given ${\mathbb {F}}\in \Gamma \text {Sch}(N,M)$, we will give a meaning to the SDE12which we will call a *Schwartz-Meyer equation*. If *X* is an *M*-valued semimartingale the second order differential  should be interpreted in local coordinates $\varphi$ as13where the first differential is an Itô differential; this expression is seen to be invariant under change of charts, thanks to the Itô formula. Then, given charts $\varphi$ in *M* and $\vartheta$ on *N*, and writing

14 $$\begin{aligned} \begin{aligned} {\mathbb {F}}(x,z)\partial _z\vartheta _\gamma&= {\mathbb {F}}_\gamma ^k(x,z) \partial _x \varphi _k + {\mathbb {F}}_\gamma ^{ij}(x,z) \partial _x^2 \varphi _{ij} \\ {\mathbb {F}}(x,z)\partial _z^2 \vartheta _{\alpha \beta }&= {\mathbb {F}}_{\alpha \beta }^k(x,z) \partial _x \varphi _k + {\mathbb {F}}_{\alpha \beta }^{ij}(x,z) \partial _x^2 \varphi _{ij} \end{aligned} \end{aligned}$$

(12) becomes the system

15 $$\begin{aligned} {\left\{ \begin{array}{ll} \text {d}X^k_t = {\mathbb {F}}^k_\gamma (X_t,Z_t) \text {d}Z_t^\gamma + \frac{1}{2} {\mathbb {F}}^k_{\alpha \beta }(X_t,Z_t) \text {d}[Z^\alpha , Z^\beta ]_t \\ \frac{1}{2} \text {d}[X^i,X^j]_t = {\mathbb {F}}^{ij}_\gamma (X_t,Z_t) \text {d}Z^\gamma _t + \frac{1}{2} {\mathbb {F}}^{ij}_{\alpha \beta }(X_t,Z_t) \text {d}[Z^\alpha ,Z^\beta ]_t \end{array}\right. } \end{aligned}$$

Computing the quadratic covariation matrix of *X* from the first equation above, using the Kunita-Watanabe identity, and comparing with the second results in the requirement that

16 $$\begin{aligned} {\mathbb {F}}^{ij}_\gamma \equiv 0; \quad {\mathbb {F}}_{\alpha \beta }^{ij} \equiv \tfrac{1}{2} \big ({\mathbb {F}}_\alpha ^i {\mathbb {F}}_\beta ^j + {\mathbb {F}}_\alpha ^j {\mathbb {F}}_\beta ^i \big ) \end{aligned}$$

which correspond precisely to the Schwartz condition (11), and justifies this requirement. (15) now reduces to its first line, i.e. the Itô SDE

17 $$\begin{aligned} \text {d}X_t^k = {\mathbb {F}}^k_\gamma (X_t,Z_t) \text {d}Z_t^\gamma + \tfrac{1}{2} {\mathbb {F}}_{\alpha \beta }^k(X_t,Z_t) \text {d}[Z^\alpha , Z^\beta ]_t \end{aligned}$$

on random intervals that make both sides of the expression well-defined.

### Example 2

(Schwartz–Meyer diffusion) Proceeding as in Example 1, but with Schwartz-Meyer equations, we can define the Schwartz-Meyer SDE18where we can call ${\mathbb {F}}_\gamma = \sigma _\gamma$ the diffusion coefficients, since they are elements of $C^{\infty }({\mathbb {R}}_{\ge 0},\Gamma TM)$; this also holds for $\gamma = 0$, but not for ${\mathbb {F}}_{\alpha \beta } \in C^{\infty }({\mathbb {R}}_{\ge 0},\Gamma {\mathbb {T}}M)$. Therefore the coefficient of $\text {d}t$, the “drift”, cannot be interpreted as a vector. Note that setting ${\mathbb {F}}_{\gamma \gamma } \equiv 0$ does not guarantee that such coefficients will vanish w.r.t. another chart, since the transformation rule for them involves the ${\mathbb {F}}_{\alpha \beta }^{ij}$’s which cannot vanish by the second Schwartz condition (16); in other words, there is no way to do away with the non vector-valued drift in (18). We can consider Schwartz Meyer diffusions as being sections of the fibre bundle

19 $$\begin{aligned} \text{ Diff}^{\, n}_\text{ Sch } M&{:}{=}&\frac{\{{\mathbb {F}}\in \text{ Sch }({\mathbb {R}}_{\ge 0} \times {\mathbb {R}}^n, M) : \forall w_1,w_2 \in {\mathbb {R}}^n \ {\mathbb {F}}(t,w_1;x) = {\mathbb {F}}(t,w_2;x) \}}{{\mathbb {F}}\sim {\mathbb {G}} \Leftrightarrow {\mathbb {F}}_{\gamma \ge 1} = {\mathbb {G}}_\gamma , \ {\mathbb {F}}_0 + \frac{1}{2} \sum _{\gamma = 1}^n {\mathbb {F}}_{\gamma \gamma } = {\mathbb {G}}_0 + \frac{1}{2} \sum _{\gamma = 1}^n {\mathbb {G}}_{\gamma \gamma }} \nonumber \\{} & {} \quad \rightarrow M \times {\mathbb {R}}_{\ge 0} \end{aligned}$$

This means that, similarly to the case of (6) we are only considering ${\mathbb {F}}$’s that do not depend explicitly on the Brownian motion, and we are quotienting out the part that is not relevant for (18).

The recent paper [1] treats SDEs on manifolds using a representation which is similar to that of (12), but which has a distinct advantage when it comes to numerical schemes. Here the authors focus on the autonomous diffusion case, without explicitly taking time as a driver ($N = {\mathbb {R}}^n$, $Z_t = W_t$), and take the field of Schwartz morphisms ${\mathbb {F}}$ to be *induced* by a *field of maps* i.e. a smooth function $f :{\mathbb {R}}^n \times M \rightarrow M$, $f_x {:}{=}f(\cdot ,x)$, s.t. for all $x \in M$, $f_x(0) = x$: this means

20 $$\begin{aligned} {\mathbb {F}}(x) = {\mathbb {T}}_0 f_x \end{aligned}$$

In coordinates $\varphi$ on *M* this amounts to

21 $$\begin{aligned} \sigma ^k_\gamma (x) = \frac{\partial (\varphi ^k \circ f_x)}{\partial w^\gamma }(0), \quad {\mathbb {F}}^k_{\alpha \beta }(x) = \frac{\partial ^2 (\varphi ^k \circ f_x)}{\partial w^\alpha \partial w^\beta } (0) \end{aligned}$$

with ${\mathbb {F}}_0 = 0$ (note how the drift comes from the quadratic variation of Brownian motion, without having to require time as a driving process). This particular form of ${\mathbb {F}}$ is useful because it automatically defines a numerical scheme for the solution of the SDE, similar to the Euler scheme, which cannot be defined in a coordinate-free way on a manifold: the linear structure lacked by *M* is replaced with iterative interpolations along the $f_x$’s. This also has the advantage of guaranteeing that if the maps are valued in *M*, so are all the approximations. “Itô-type” Diffusions on manifolds have also been investigated by other authors, most notably by [3, Ch.4] (although we refer to the more recent exposition [9, §7.2]), who call the bundle $\text {Diff}_\text {Sch}^{\, n} M$ the *Itô bundle*, and give a local description of it. For a comparison of the Schwartz-Meyer and Itô bundle approaches, we refer the reader to [8, Ch. 1].

There is a way of writing Itô equations on a manifold so that all the coefficients, drift included, are vectors. It involves considering the additional structure of a linear connection $\nabla$ on *M*, i.e. a covariant derivative

22 $$\begin{aligned} \nabla :TM \times \Gamma TM \rightarrow TM \end{aligned}$$

which is a smooth function that maps $T_xM \times \Gamma TM$ to $T_xM$, is ${\mathbb {R}}$-bilinear, and satisfies the Leibniz rule $\nabla _{U_x}(f V) = f(x) \nabla _{U_x}V + (U_xf)V_x$. Equivalently, a connection is described through its Hessian

23 $$\begin{aligned} \nabla ^2 :C^\infty M \rightarrow \Gamma (T^*M \otimes T^*M) \end{aligned}$$

which is an ${\mathbb {R}}$-linear map satisfying $\nabla ^2(fg) = f \nabla ^2 g + g \nabla ^2 f + \text {d}f \otimes \text {d}g + \text {d}g \otimes \text {d}f$ for all $f, g \in C^\infty M$. These two data are equivalent and related by

24 $$\begin{aligned} \langle \nabla ^2_x f, V \otimes U \rangle = U_x(Vf) - (\nabla _{U_x}V) f \end{aligned}$$

If $\Gamma ^{ij}_k$ are the Christoffel symbols of $\nabla$ w.r.t. a chart $\varphi$ (this means $\nabla _{\partial _x \varphi _i}\partial \varphi _j = \Gamma ^k_{ij}(x) \partial _x \varphi _k$), the Hessian can be written as

25 $$\begin{aligned} \nabla ^2_x f = (\partial ^2_x \varphi _{ij} - \Gamma _{ij}^k(x) \partial _x \varphi _k)(f) \text {d}_x \varphi ^i \otimes \text {d}_x \varphi ^j \end{aligned}$$

We will only be interested in connections modulo torsion, so it is not limiting for us to assume that a connection is symmetric or torsion-free, i.e. that its torsion tensor $\langle \tau _\nabla , U \otimes V \rangle = \nabla _U V - \nabla _V U - [U,V]$ vanishes, or equivalently that its Hessian is valued in $\Gamma (T^*M \odot T^*M)$. By far the most important example of such a connection is the Levi-Civita connection of a Riemannian metric $\mathcalligra {g}$; in this case the Hessian takes the form $\langle \nabla ^2_x f, U_x \otimes V_x \rangle = \mathcalligra {g}( \nabla _{U_x} \text {grad}^{\mathcalligra {g}}f, V_x )$. Torsion-free connections are relevant to our study of SDEs in that they correspond to the splittings of (9), i.e. a linear left inverse $\mathcalligra {q}$ to $\mathcalligra {i}$ or a linear right inverse $\mathcalligra {j}$ to $\mathcalligra {p}$
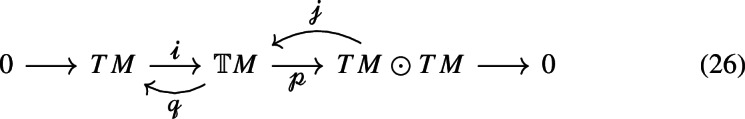
 The existence of the bundle maps $\mathcalligra {j}$ and $\mathcalligra {q}$ are equivalent to one another and to the isomorphism $(\mathcalligra {q}, \mathcalligra {p}) :{\mathbb {T}}M \rightarrow TM \oplus (TM \odot TM)$ (this is the well-known splitting lemma [10, p.147], valid in the category of vector bundles). A torsion-free connection $\nabla$ on *M* is equivalent to a splitting by setting

27 $$\begin{aligned} (\mathcalligra {q}_x L_x)f {:}{=}L_xf - \langle \nabla ^2_x f, \mathcalligra {p}_xL_x \rangle \end{aligned}$$

We recall that, given $V \in \Gamma TM$, the “composition” $U_x(V) \in {\mathbb {T}}_x M$ is defined by $U_x(V)f {:}{=}U_x(y \mapsto V_y f)$, and we have

28 $$\begin{aligned} {\mathcalligra {p}_x (U_x(V)) = U_x \odot V_x, \quad {\mathcalligra {q}}_x (U_x(V)) = \nabla _{U_x} V } \end{aligned}$$

Using that $\partial ^2_x \varphi _{ij} = \partial _x \varphi _i (\partial \varphi _j)$ and (25) we have

29 $$\begin{aligned} {\mathcalligra {p}_x \partial ^2_x\varphi _{ij} = \partial _x \varphi _i \odot \partial _x \varphi _j, \quad {\mathcalligra {q}}_x \partial ^2_x\varphi _{ij} = \Gamma _{ij}^k(x) \partial _x \varphi _k } \end{aligned}$$

Another way to view this correspondence is by .

Now, given symmetric connections on *N* and *M*, a field of Schwartz morphisms ${\mathbb {F}}\in \Gamma \text {Sch}(N,M)$ can be viewed as a field of block matrices

30 $$\begin{aligned} \begin{bmatrix} F &{} G \\ 0 &{} F \otimes F \end{bmatrix}(x,z) :T_zN \oplus (T_zN \odot T_zN) \rightarrow T_xM \oplus (T_xM \odot T_xM) \end{aligned}$$

One can then require that $G \equiv 0$, so that ${\mathbb {F}}$ reduces to *F*, which defines the *Itô equation*

31 $$\begin{aligned} \text {d}X_t = F(X_t,Z_t) \text {d}Z_t \end{aligned}$$

Such equations have been considered in [7]. The data needed to define this equation is the same as that involved in the definition of the Stratonovich equation (4), namely an element of $\Gamma \text {Hom}(TN,TM)$, but the meaning of the equation depends on the connections on *N* and *M*. In local coordinates, using (29) to specify ${\mathbb {F}}_{\alpha \beta }^k$ in (17) to the case $G \equiv 0$, this equation takes the form

32 $$\begin{aligned}&\text {d}X_t^k = F^k_\gamma (X_t,Z_t) \text {d}Z_t^\gamma + \tfrac{1}{2} \big ( {^N\!}\Gamma _{\alpha \beta }^\gamma (Z_t) F^k_\gamma (X_t,Z_t)\nonumber \\ {}&\quad - {^M\!}\Gamma _{ij}^k(X_t) F^i_\alpha F^j_\beta (X_t,Z_t) \big ) \text {d}[Z^\alpha , Z^\beta ]_t \end{aligned}$$

Recall that an $(M,\nabla )$-valued semimartingale is a *local martingale* if for all $f \in C^\infty M$33is a real-valued local martingale (the integral is to be interpreted as half the quadratic variation of *X* along the bilinear form $\nabla ^2 f$); this property coincides with the usual local martingale property when *M* is a vector space. In local coordinates an application of (25) and (13) shows that the local martingale property corresponds to the requirement that

34 $$\begin{aligned} \text {d}X^k_t + \tfrac{1}{2} \Gamma ^k_{ij}(X_t) \text {d}[X^i,X^j]_t \end{aligned}$$

be a real-valued local martingale for each *k*.

In the following example we examine the case of diffusions, defined using Itô equations, in which the issue of the drift not being a vector is (partially) resolved:

### Example 3

(Itô diffusion) Example 2 specified to the above case (*M* has a symmetric connection, $G \equiv 0$ in (30)) becomes the equation

35 $$\begin{aligned} \text {d}X_t = \sigma _\gamma (X_t,t) \text {d}W^\gamma _t + \mu (X_t,t) \text {d}t \end{aligned}$$

where now $\mu (x,t) = {\mathbb {F}}(x,t) \in T_xM$ can legitimately be referred to as the “drift vector”. Note however that in an arbitrary chart $\varphi$ the drift will still carry a correction term:

36 $$\begin{aligned} \text {d}X_t^k = \sigma ^k_\gamma (X_t,t) \text {d}W_t^\gamma + \bigg ( \mu ^k(X_t,t) - \frac{1}{2} \sum _{\gamma = 1}^n \Gamma _{ij}^k(X_t) \sigma ^i_\gamma \sigma ^j_\gamma (X_t,t) \bigg ) \text {d}t \end{aligned}$$

which reduces to the ordinary Itô lemma if $M = {\mathbb {R}}^m$ and the chart $\varphi$ is a diffeomorphism of ${\mathbb {R}}^m$. The ${^N\!}\Gamma _{\alpha \beta }^\gamma$’s do not appear since the driver is already valued in a Euclidean space. The data needed to define such an equation coincides with that needed for (4), so we can define the bundle

37 $$\begin{aligned} \text {Diff}_\text {Ito}^{\, n} M {:}{=}\text {Diff}_\text {Strat}^{\, n} M \rightarrow M \times {\mathbb {R}}_{\ge 0} \end{aligned}$$

already defined in (6). Crucially, however, the Stratonovich and Itô calculi give different meanings to the equation defined by a section of this bundle; in particular, a torsion-free connection on *M* is required in the latter case. The “Itô” and “Strat” therefore do not represent differences in the bundles, which are identical, but only serve as a reminder of which calculus is being used to give the section the meaning of an SDE.

Itô equations on manifolds are the true generalisation of their Euclidean space-valued counterparts, but have the disadvantage of only being defined w.r.t. a specific connection. For instance, if $F \in \Gamma \text {Diff}^{\, n}_{\text {Ito}}$, *M* is Riemannian with $M'$ a Riemannian submanifold s.t. for all *z* and $x \in M'$, *F*(*z*, *x*) maps to $T_xM'$, *F* does not in general define an Itô equation on $M'$, since the Riemannian connection on $M'$ is not in general the restriction of that of *M*. However, *F*, seen as a field of Schwartz morphisms, does define a Schwartz-Meyer equation on $M'$ (with a *G* term that is in general non-zero w.r.t. to the Riemannian connection on $M'$).

In the following table we summarise the advantages of these three ways of representing SDEs on manifolds:

**Table:** 

	Stratonovich	Schwartz-Meyer/2-jet	Itô
Does not require $\nabla$	$\checkmark$	$\checkmark$	
Uses Itô integration		$\checkmark$	$\checkmark$
Coefficients are vectors	$\checkmark$		$\checkmark$

It is natural to ask how these three types of equations are related to one another. In the case of diffusions, there exists a commutative diagram of bijections 
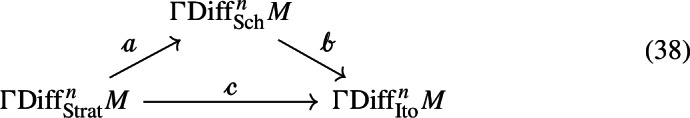
 All three ${\mathcalligra {a}}, {\mathcalligra {b}}, {\mathcalligra {c}}$ are the identity on the diffusion coefficients. The behaviour of ${\mathcalligra {a}}, {\mathcalligra {b}}, {\mathcalligra {c}}$ on the Stratonovich, Schwartz-Meyer and Itô drifts is explained below

39 $$\begin{aligned} {\mathcalligra {a} b {:}{=}b + \frac{1}{2} \sum _{\gamma = 1}^n \sigma _\gamma (\sigma _\gamma ), \quad {\mathcalligra {b}} \eta {:}{=}{\mathcalligra {q}} \eta , \quad {\mathcalligra {c}} b {:}{=}b + \frac{1}{2} \sum _{\gamma = 1}^n \nabla _{\sigma _\gamma } \sigma _\gamma } \end{aligned}$$

Note that, while ${\mathcalligra {b}}$ and ${\mathcalligra {c}}$ depend on the connection, ${\mathcalligra {a}}$ does not. If $\eta = {\mathbb {F}}_0 + \frac{1}{2} \sum _{\gamma = 1}^n {\mathbb {F}}_{\gamma \gamma }$ is a Schwartz-Meyer drift, (11) and (28) force $\eta - \frac{1}{2}\sum _{\gamma = 1}^n \sigma _\gamma (\sigma _\gamma )$ to lie in $T_x M$, which is thus ${\mathcalligra {a}}^{-1} \eta$. Moreover, we have ${\mathcalligra {b}}^{-1} \mu = {\mathcalligra {i}} \mu + \frac{1}{2} \sum _{\gamma = 1}^n {\mathcalligra {j}} (\sigma _\gamma \odot \sigma _\gamma )$ and ${\mathcalligra {c}}^{-1} \mu = b - \frac{1}{2} \sum _{\gamma = 1}^n \nabla _{\sigma _\gamma } \sigma _\gamma$. ${\mathcalligra {a}}, {\mathcalligra {b}}, {\mathcalligra {c}}$ define correspondences of SDEs in the sense that solutions are preserved (e.g. *X* is a solution of $F \in \text {Diff}_\text {Strat}^{\, n} M$ if and only if *X* is a solution of ${\mathcalligra {a}} F$, and the same for ${\mathcalligra {b}}, {\mathcalligra {c}}$). This is immediate by the expression of such equations in charts, by (28) and the usual Itô-Stratonovich conversion formula.

### Remark 1

What makes Itô-Stratonovich conversion formulae difficult to state in the case of a general manifold-valued semimartingale driver *Z*, is that the change of calculus involves the emergence of new drivers which are not naturally valued in the manifold where Z is valued (the quadratic covariation of *Z*). Nevertheless, the map ${\mathcalligra {a}}$ can be defined in this general setting [6, Lemma 7.22], though its inverse cannot canonically.

## Manifolds embedded in Euclidean space

In this paper we will mostly be concerned with manifolds embedded in ${\mathbb {R}}^d$: these can be studied using the extrinsic, canonical, ${\mathbb {R}}^d$-coordinates instead of non-canonical local ones. Let *M* be an *m*-dimensional smooth manifold embedded in ${\mathbb {R}}^d$. We assume *M* to be locally given by a non-degenerate Cartesian equation $F(x) = 0$: *M* can be described globally in this way if and only if it is closed and its embedding has trivial normal bundle; therefore, to preserve generality, we only assume *F* to be local. Throughout this paper the letter *x* will denote a point in ${\mathbb {R}}^d$ and the letter *y* a point in *M*. Thus $F :{\mathbb {R}}^d \rightarrow {\mathbb {R}}^{d-m}$ is a submersion, which implies is an invertible $(d-m)\times (d-m)$ matrix for all $x \in {\mathbb {R}}^d$ ($JF(x) \in {\mathbb {R}}^{(d-m) \times d}$ the Jacobian of *F* at *x*):

40 $$\begin{aligned} {\begin{matrix}{} JF(x)JF(x)^\intercal v^\intercal = 0 \ {} &{}\Rightarrow \ (vJF(x))(vJF(x))^\intercal = v JF(x)JF(x)^\intercal v^\intercal = 0 \ \\ {} &{}\Rightarrow \ v = 0 \end{matrix}}\end{aligned}$$

Let $\pi$, defined on a tubular neighbourhood *T* of *M* in ${\mathbb {R}}^d$ be the Riemannian submersion

41 $$\begin{aligned} \pi (x) {:}{=}\arg \min \{ |x - y| : y \in M \} \end{aligned}$$

This map can be seen to exist by using the normal exponential map defined in [14, p.132], and is constant on the affine $(d-m)$-dimensional slices of *T* which intersect *M* orthogonally: this is because the fibre $\pi ^{-1}(y)$ coincides with the union of all geodesics in ${\mathbb {R}}^d$ (i.e. straight line segments) which start at *y*, with initial velocity orthogonal to *M*, each taken for *t* in some open interval containing 0. It is important also to remember that $\pi$ is unique given the embedding of *M* (on a thin enough *T* such that it is well defined), whereas *F* is not canonically determined. In what follows we will be concerned with understanding which quantities are dependent on the chosen *F* and which instead only depend on the embedding of *M*. The only properties of $\pi$ that we will need are that

42 $$\begin{aligned} F \circ \pi \equiv 0, \quad \pi |_M = \mathbbm {1}_M \Rightarrow \pi \circ \pi \equiv \pi \end{aligned}$$

Differentiating these (the second up to order 2) we obtain

43 $$\begin{aligned} \begin{aligned} \frac{\partial F}{\partial x^h}(\pi (x)) \frac{\partial \pi ^h}{\partial x^k}(x)&= 0 \\ \frac{\partial \pi }{\partial x^h}(\pi (x)) \frac{\partial \pi ^h}{\partial x^k}(x)&= \frac{\partial \pi }{\partial x^k}(x) \\ \frac{\partial ^2 \pi }{\partial x^a \partial x^b}(\pi (x)) \frac{\partial \pi ^a}{\partial x^i} \frac{\partial \pi ^b}{\partial x^j}(x) + \frac{\partial \pi }{\partial x^h}(\pi (x)) \frac{\partial ^2 \pi ^h}{\partial x^i \partial x^j}(x)&= \frac{\partial ^2 \pi }{\partial x^i \partial x^j}(x) \end{aligned} \end{aligned}$$

If $V_y \in T_M$ and *X* is a smooth curve s.t. $X_0 = y$ and $\dot{X}_0 = V(y)$, differentiating $\pi (X_t) = X_t$ results in $J\pi (y) = V_y$: this shows that $J\pi |_{TM} = \mathbbm {1}_{TM}$. By a similar argument, the fact that $\pi ^{-1}(y)$ is a straight line segment that intersects *M* orthogonally implies that $J\pi |_{T^\bot M} = \mathbbm {1}_{T^\bot M}$ ($T^\bot _y M$ the normal bundle of *M* at *y*). These two statements mean that

44 $$\begin{aligned} P(y) = J\pi (y) \quad \text {for } y \in M \end{aligned}$$

where $P(y) :T_y {\mathbb {R}}^d \rightarrow T_yM$ is the orthogonal projection onto the tangent bundle of *M*, which can be defined in terms of *F* as

45 $$\begin{aligned} \begin{aligned} P(x)&{:}{=}\mathbbm {1} - Q(x) \qquad \text {where}\\ Q(x)&{:}{=}JF^\intercal (x)(JF(x)JF^\intercal (x))^{-1}JF(x) \in {\mathbb {R}}^{d\times d} \qquad \text {and we have} \\ PQ(x)&= 0 = QP(x), \quad QQ(x) = Q(x) = Q^\intercal (x), \quad PP(x) = P(x)= P^\intercal (x) \end{aligned} \end{aligned}$$

The notation is borrowed from [5]. Note that we can use *F* to define *P*, *Q* on a tubular neighbourhood of *M*, but these will only be independent of *F* on *M*. $Q(y):T_y {\mathbb {R}}^d \rightarrow T_y^\bot M$ is the orthogonal projection onto the normal bundle. Another consequence of (43) (evaluated at $y \in M$) that will be useful is that, for $V_y, W_y \in T_y{\mathbb {R}}^d$, and denoting  (with $U = V,W$)46Actually, to show that the third term statement in the first line, we need a separate argument:

### Remark 2

Let $$U \subseteq {\mathbb {R}}^d$$, $$f :U \rightarrow {\mathbb {R}}^e$$, $$y \in U$$, $$A_y,B_y \in T_y{\mathbb {R}}^d$$. Then

47 $$\begin{aligned} \frac{\partial ^2 f}{\partial x^i \partial x^j}(y) A^i_y B^j_y \end{aligned}$$

only depends on *f* restricted to the affine plane (or line) centred in *y* and spanned by $A_y,B_y$. Indeed, intending with *A* the extension of $A_y$ to a constant vector field on *U*, we can write

48 $$\begin{aligned} \frac{\partial ^2 f}{\partial x^i \partial x^j}(y) A^i_y B^j_y = \frac{\partial }{\partial x^j} \bigg |_y \bigg ( \underbrace{\frac{\partial f}{\partial x^i}(x) A^i_x}_{{=}{:}g(x)} \bigg ) B^j_y \end{aligned}$$

This is the directional derivative of *g* at *y* in the direction $B_y$, and therefore only depends on the restriction of *g* to the affine line $\text {span}\{B_y\}$. But *g*(*x*) is itself a directional derivative, and only depends on *f* restricted to the affine line $\text {span}\{ A_x \}$. Thus the whole expression only depends on *f* restricted to $\bigcup _{x \in \text {span}\{B_y\} } \text {span}\{ A_{x}\} = \text {span}\{ A_y, B_y\}$.

This shows that the term in question only depends on $\pi$ restricted to , which is the constant *y* map, whose derivatives therefore vanish.

### Remark 3

The other terms appearing in (46) have a description that should be more familiar to differential geometers:49where ${^{{\mathbb {R}}^d}}\nabla$ denotes covariant differentiation in ${\mathbb {R}}^d$ (i.e. just directional differentiation). Notice this is true independently of the chosen extension of  to local vector fields, a priori needed to give the RHSs a meaning. The first term is the second fundamental form of ${\overline{V}}_y, {\overline{W}}_y$ [13, p.134], whereas the second term is the second fundamental tensor [11, Def. 3.6.1]. If *M* is an open set of an affine subspace of *M*, $\pi$ is a linear map and both terms vanish. We prove the first of the two equalities in (49), the second is proved similarly:

50 $$\begin{aligned} Q(y){^{{\mathbb {R}}^d}\nabla _{{\overline{V}}_y}} {\overline{W}} = Q_j(y) \frac{\partial {\overline{W}}^j}{\partial x^i}(y) {\overline{V}}^i_y = -\frac{\partial Q_j}{\partial x^i}(y) {\overline{W}}^j_y {\overline{V}}^i_y = \frac{\partial ^2 \pi }{\partial x^i \partial x^j}(y){\overline{V}}^i_y {\overline{W}}^j_y \end{aligned}$$

where the second equality follows from the fact that $Q {\overline{W}} = 0$ (and that the derivative is taken in a tangential direction, i.e. ${\overline{V}}_y \in T_y M$), and the last equality is given by (53) below. Note that the terms of (49) are extrinsic, in the sense that they depend on the embedding of *M*, unlike

51 $$\begin{aligned} {^M \!}\nabla _{{\overline{V}}_y} {\overline{W}}_y = P(y) {^{{\mathbb {R}}^d}\nabla _{{\overline{V}}_y}} {\overline{W}} \end{aligned}$$

the Levi-Civita connection of the Riemannian metric on *M*, which is intrinsic to *M*.

Finally, it will be necessary to consider the relationship between the derivatives of *P*, *Q* and the second derivatives of $\pi$. We differentiate (44) at time 0 along a smooth curve $Y_t$ in *M* with $Y_0 = 0$ and $\dot{Y}_0 = {\overline{V}}_y \in T_y M$ and obtain

52 $$\begin{aligned} \frac{\partial P_k}{\partial x^h}(y) {\overline{V}}^h_y = \frac{\partial ^2 \pi }{\partial x^i \partial x^j}(y) {\overline{V}}^i_y \end{aligned}$$

from which we obtain, for $W \in T_yM$53where we have used (46).

We now consider a setup ${\mathcal {S}} = (\Omega , {\mathcal {F}}, P)$ satisfying the usual conditions, *W* an *n*-dimensional Brownian motion defined on ${\mathcal {S}}$. Consider the *W*-driven diffusion Stratonovich SDE

54 $$\begin{aligned} \text {d}X_t^k = \sigma _\gamma ^k(X_t,t) \circ \text {d}W_t^\gamma + b^k(X_t,t) \text {d}t, \quad X_0 = y_0 \in M \end{aligned}$$

As already discussed in Sect. 2, the natural condition on $\sigma _\gamma , b$ which guarantees that *X* will stay on *M* for its lifetime is their tangency to *M*:

55 $$\begin{aligned} Q(y) \sigma _\gamma (y,t) = 0 = Q(y) b(y,t) \quad \text {for all } y \in M, t \ge 0, \gamma = 1,\ldots , n \end{aligned}$$

Our focus, however, will be mostly on the Itô SDE

56 $$\begin{aligned} \text {d}X_t^k = \sigma ^k_\gamma (X_t,t) \text {d}W^\gamma _t + \mu ^k(X_t,t) \text {d}t, \quad X_0 = y_0 \in M \end{aligned}$$

with smooth coefficients defined in $[0,+\infty ) \times {\mathbb {R}}^d$; we do not assume them to be globally Lipschitz, so the solution might only exist up to a positive stopping time, not in general bounded from below by a positive deterministic constant. We are interested in deriving the “tangency condition” for the above SDE, i.e. a condition on the coefficients that will guarantee that the solution will not leave *M*. One way to impose this is to convert (56) to Stratonovich form

57 $$\begin{aligned} \text {d}X_t^k = \sigma ^k_\gamma (X_t,t) \circ \text {d}W^\gamma _t + \bigg (\mu ^k - \frac{1}{2} \sum _{\gamma = 1}^n \sigma ^h_\gamma \frac{\partial \sigma ^k_\gamma }{\partial x^h} \bigg )(X_t,t) \text {d}t, \quad X_0 = y_0 \in M \end{aligned}$$

and require (55):

58 $$\begin{aligned} {\left\{ \begin{array}{ll} Q_k(y) \sigma ^k_\gamma (y,t) = 0 \\ Q_k(y) \bigg ( \mu ^k - \frac{1}{2} \sum _{\gamma = 1}^n \sigma ^h_\gamma \frac{\partial \sigma ^k_\gamma }{\partial x^h} \bigg )(y,t) = 0 \end{array}\right. } \end{aligned}$$

Now, given that $Q \sigma _\alpha$ vanishes on *M*, all its directional derivatives along the tangent directions $\sigma _\beta$ will too, which gives, using (53)

59 $$\begin{aligned} 0 = \frac{\partial (Q \sigma _\alpha )}{\partial x^h} \sigma _\beta ^h = \frac{\partial Q_i}{\partial x^j} \sigma ^i_\alpha \sigma ^j_\beta + Q_k \frac{\partial \sigma ^k_\alpha }{\partial x^h} \sigma ^h_\beta \Longrightarrow Q_k \frac{\partial \sigma ^k_\alpha }{\partial x^h} \sigma ^h_\beta = \frac{\partial ^2 \pi }{\partial x^i \partial x^j} \sigma _\alpha ^i \sigma _\beta ^j \quad \text {on } M \nonumber \\ \end{aligned}$$

We can thus reformulate the second equation in (58) to obtain

60 $$\begin{aligned} {\left\{ \begin{array}{ll} Q_k(y) \sigma ^k_\gamma (y,t) = 0 \\ Q_k(y) \mu ^k(y,t) = \frac{1}{2} \sum _{\gamma = 1}^n \frac{\partial ^2 \pi }{\partial x^i \partial x^j}(y) \sigma ^i_\gamma \sigma ^j_\gamma (y,t) \end{array}\right. } \end{aligned}$$

This is useful because it removes the reliance of this constraint on the derivatives of $\sigma$, and can be interpreted as saying that the diffusion coefficients must be tangent to *M* and the Itô drift must instead lie on the space parallel to the tangent space of *M*, displaced by an amount which depends on the second fundamental form of *M* applied to the diffusion coefficients.

### Remark 4

(Tangency of a second-order differential operator) (60) can also be derived by writing the second order tangency condition for $L_y^k \partial _y x_k + L^{ij}_y \partial ^2_y x_{ij} = L_y \in {\mathbb {T}}_y {\mathbb {R}}^d$ to belong to ${\mathbb {T}}_y M$: this is done by writing ${\mathbb {T}}_y \pi L_y = L_y$ in ${\mathbb {R}}^d$-coordinates as

61 $$\begin{aligned} \begin{bmatrix} L^h_y \\ L^{ab}_y \end{bmatrix} = \begin{bmatrix} \frac{\partial \pi ^h}{\partial x^k} &{} \frac{\partial ^2 \pi ^h}{\partial x^i \partial x^j} \\ 0 &{} \frac{\partial \pi ^a}{\partial x^i} \frac{\partial \pi ^b}{\partial x^j} \end{bmatrix}(y) \begin{bmatrix} L^k_y \\ L^{ij}_y \end{bmatrix} \end{aligned}$$

and then applying it to $L_y = \sigma _\gamma (y,t), \eta (y,t)$, given in terms a field of Schwartz morphisms ${\mathbb {F}}$ as

62 $$\begin{aligned} \sigma _\gamma ^k = {\mathbb {F}}_\gamma ^k, \quad \eta ^k = {\mathbb {F}}_0^k + \frac{1}{2} \sum _{\gamma = 1}^n {\mathbb {F}}^k_{\gamma \gamma } \end{aligned}$$

Note that it would instead be incorrect to split ${\mathbb {F}}$ according to the Euclidean connection into a matrix with *F* and *G* terms as in (30), and then to require that *F* and *G* map to *TM*, since the splitting of ${\mathbb {F}}$ according to the connection on *M* will be different, i.e. the diagram 
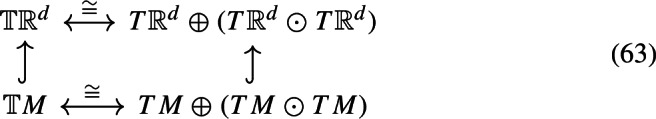
 does not commute.

We now compute the Hessian for embedded *M*: for $f \in C^\infty M$ we have

64 $$\begin{aligned} \big \langle {^M \!} \nabla ^2_y f, {\overline{V}}_y \otimes {\overline{U}}_y \big \rangle = \big \langle {^{{\mathbb {R}}^d} \!} \nabla ^2_y (f \circ \pi ), {\overline{V}}_y \otimes {\overline{U}}_y \big \rangle \end{aligned}$$

where we have used (24), (51) to reduce this to a computation of directional derivatives, and finally (53) (the argument is similar to (50)). ${^{{\mathbb {R}}^d} \!} \nabla ^2$ of course is just the ordinary Hessian. We can now compute ${^M \!\!} {\mathcalligra {q}}$, the splitting appearing in (26) w.r.t. the connection on *M*: if ${\mathbb {T}}_yM \ni L_y = L_y^k \partial _y x_k + L^{ij}_y \partial ^2_y x_{ij}$, using (27) yields65which means66Therefore the condition on an arbitrary Schwartz morphism of being Itô w.r.t. to the Riemannian connection on *M* in the sense of Example 3 is ${^M \!\!}{\mathcalligra {q}} \circ {\mathbb {F}}\circ {^{{\mathbb {R}}^d} \!\!}\mathcalligra {j} = 0$, or ${^M \!\!}\mathcalligra {q} {\mathbb {F}}_{\alpha \beta } = 0$, which in ${\mathbb {R}}^d$-coordinates is

67 $$\begin{aligned} P_k(y) {\mathbb {F}}_{\alpha \beta }^k(y,t) = 0 \end{aligned}$$

Compare this with the stronger condition of ${\mathbb {F}}$ of being Itô w.r.t. to the connection on ${\mathbb {R}}^d$, which is ${\mathbb {F}}_{\alpha \beta }^k(y,t) = 0$. Thus, given an Itô equation ${\mathbb {F}}$ on *M*, defined as in (35) ($\sigma _\gamma = {\mathbb {F}}_\gamma$, ${\overline{\mu }} = {\mathbb {F}}_0$) we have that the drift in ${\mathbb {R}}^d$ of such equation is given by ${\overline{\mu }}^k + \frac{1}{2} \sum _{\gamma = 1}^n {\mathbb {F}}^k_{\gamma \gamma }$, with the first term tangent to *M* and the second orthogonal to *M*, and equal to $\frac{1}{2} \sum _{\gamma = 1}^n \frac{\partial \pi ^h}{\partial x^i \partial x^j} \sigma ^i \sigma ^j_\gamma$, by Remark 4 and (67). Therefore an Itô equation on *M* with coefficients $\sigma _\gamma , {\overline{\mu }}$ is read in ambient coordinates as

68 $$\begin{aligned} \text {d}X_t^k = \sigma _\gamma ^k(Y_t,t) \text {d}W_t^\gamma + \bigg ({\overline{\mu }}^k + \frac{1}{2} \sum _{\gamma = 1}^n \frac{\partial ^2 \pi ^k}{\partial x^i \partial x^j} \sigma ^i_\gamma \sigma ^j_\gamma \bigg )(Y_t,t) \text {d}t \end{aligned}$$

Notice that the tangential part of the ${\mathbb {R}}^d$-drift, ${\overline{\mu }}$, is arbitrary, while its orthogonal part is determined by the diffusion coefficients, and the condition that the solution remain on *M*.

**Fig. 1:**
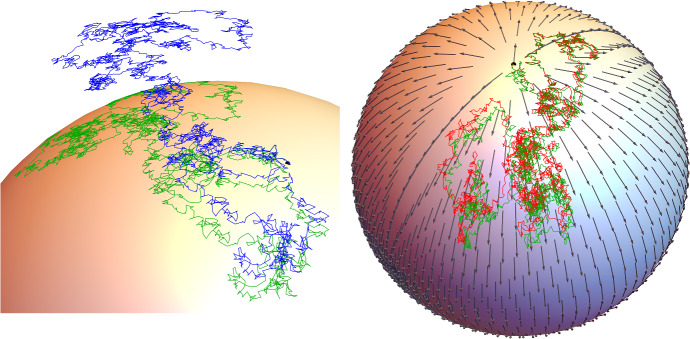
On the left a sample path of the solution to the Itô equation (blue) with the two diffusion coefficients $2(x^2+y^2+z^2)^{-1}(-y,x,0)$, $2(x^2+y^2+z^2)^{-1}(0,-z,y)$, which are tangent to $S^2 \hookrightarrow {\mathbb {R}}^3$, zero drift and initial condition (0, 1, 0); in the same plot a sample path (using the same random seed) of the solution to the Stratonovich equation (green) defined by the same vector fields and initial condition. The solution to the Itô equation drifts radially outwards, while the solution to the Stratonovich equation remains on $S^2$. On the right we compare the same Stratonovich path with a sample path of the solution to the Itô equation (red) with the same diffusion coefficients and initial condition, but with the orthogonal drift term necessary to keep the solution on $S^2$ (60). The resulting solution is an $S^2$-valued local martingale, while the solution to the Stratonovich equation is not: this is illustrated by plotting the vector field on $S^2$ given by tangential component of the Itô drift possessed by the Stratonovich equation: this can be viewed as a manifold-valued drift component

The notion of *M*-valued local martingale also has a description in terms of ambient coordinates [6, Par. 4.10]: for an *M*-valued Itô process (such as the solution to (68)) the local martingale property is equivalent to requiring that the drift be orthogonal to *M* at each point (and thus determined by the diffusion coefficients; for (68) this means ${\overline{\mu }} = 0$). This condition is very reminiscent of the property of geodesics of having acceleration orthogonal to *M* [13, Lemma 8.5].

Using all (39) and (66) it is easy to verify that converting between Stratonovich, Schwartz–Meyer and Itô equations on *M* is equivalent when treating the equations as being valued in *M* or in ${\mathbb {R}}^d$. By this we mean that, denoting with $\text {Diff}_{\text {Strat},M}^{\, n} {\mathbb {R}}^d$ the bundle of Stratonovich equations on ${\mathbb {R}}^d$ which restrict to equations on *M* (and analogously for the other two diffusion bundles) the maps $\mathcalligra {a}, \mathcalligra {b}, \mathcalligra {c}$ of (38) fit into the commutative diagram
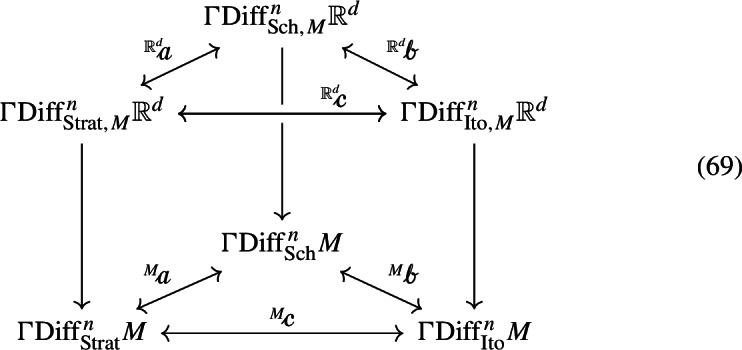


where vertical arrows denote restriction. An embedding argument immediately allows us to extend this assertion to the case where ${\mathbb {R}}^d$ is substituted with a Riemannian manifold of which *M* is a Riemannian submanifold. This confirms there is no ambiguity in converting an *M*-valued SDE between its various forms.

### Example 4

(Time dependent submanifold) Observe that the tangency conditions (55) and (60) can be written respectively as70for *any* smooth map ${\widetilde{\pi }}$ defined on a tubular neighbourhood of *M*, with values in *M*, s.t. ${\widetilde{\pi }}|_M =$, by the same exact reasoning (for the Itô case we argue as in Remark 4). $J {\widetilde{\pi }}(y)$ is no longer the orthogonal projection *P*(*y*), but still restricts to the identity on $T_yM$ for $y \in M$, i.e. it has the property that $\ker ( {\widetilde{\pi }}) = TM$ on *M*. Allowing ourselves to consider all such tubular neighbourhood projections is useful in the following application. Given that we are considering time-dependent equations, it is very natural to also allow the submanifold *M* to be time-dependent. Making this precise entails considering a smooth $(m+1)$-dimensional manifold ${\widetilde{M}}$ embedded in ${\mathbb {R}}^{1+d}$, s.t. $M_t {:}{=}{\widetilde{M}} \cap \{x_0 = t\}$ is a smooth *m*-dimensional manifold embedded in $\{x_0 = t\} \times {\mathbb {R}}^d$. We are looking for conditions on $\sigma , b$ (resp. $\mu$) which are sufficient to guarantee the solution to (54) (resp. (56)) $X_t$ to belong to $M_t$ for all *t* for which it is defined. We then consider the ${\mathbb {R}}^{1+d}$-valued process $(t,X_t)$, which satisfies the dynamics

71 $$\begin{aligned} \text {d}\begin{bmatrix} t \\ X_t \end{bmatrix} = \begin{bmatrix} 0 \\ \sigma (X_t,t) \end{bmatrix} \circ \text {d}W_t + \begin{bmatrix} 1 \\ b(X_t,t) \end{bmatrix} \text {d}t \quad \text {resp.} \quad =\begin{bmatrix} 0 \\ \sigma (X_t,t) \end{bmatrix} \text {d}W_t + \begin{bmatrix} 1 \\ \mu (X_t,t) \end{bmatrix} \text {d}t \quad \quad \end{aligned}$$

Then, given a thin enough tubular neighbourhood of ${\widetilde{M}}$ in ${\mathbb {R}}^{1+d}$ consider the map

72 $$\begin{aligned} {\widetilde{\pi }} :{\widetilde{T}} \rightarrow {\widetilde{M}},\quad {\widetilde{\pi }} (t,x) = \pi _t(x) \end{aligned}$$

where $\pi _t$ is defined as in (41) for the manifold $M_t$. Notice that this does not in general coincide with the Riemannian projection of a tubular neighbourhood onto ${\widetilde{M}}$, which in general has no reason to preserve time, i.e. be expressible as a union of $\pi _t$’s. The identity $J {\widetilde{\pi }} J {\widetilde{\pi }} = J {\widetilde{\pi }}$ can be written in block matrix form as

73 $$\begin{aligned} \left[ \begin{array}{c|c c c} 1 &{} 0 &{} \cdots &{} 0 \\ \hline \\ J\pi _t {\dot{\pi }}_t + {\dot{\pi }}_t &{} &{} J\pi _t J\pi _t &{} \\ \end{array} \right] = \left[ \begin{array}{c|c c c} 1 &{} 0 &{} \cdots &{} 0 \\ \hline \\ {\dot{\pi }}_t &{} &{} J\pi _t &{} \\ \end{array} \right] \end{aligned}$$

where we are denoting ${\dot{\pi }}_t(y) = \frac{\text {d}}{\text {d}t} \pi _t(y)$: this implies that at each point $y \in M_t$, ${\dot{\pi }}_t(y) \in T_y^\bot M_t$. This choice of the tubular neighbourhood projection will be further motivated later on, in Example 5, Example 6. In view of the above considerations, we can use it anyway to impose tangency of the SDE: this results in an unmodified condition on the diffusion coefficients, and the conditions on the orthogonal components of the Stratonovich and Itô drifts are given respectively by74which keep track of the evolution of $M_t$ in time.

## Projecting SDEs

In Sect. 2 we discussed three ways of representing SDEs on manifolds: Stratonovich, Schwartz-Meyer and Itô. In this section we will define, for each one of these representations, a natural projection of the SDE onto a submanifold. We will mostly take the ambient manifold to be ${\mathbb {R}}^d$, which will allow us to use the theory of the previous section to derive formulae for the projections in ambient coordinates.

Let *M* be a smooth submanifold of the smooth manifold *D*, let *T* be a tubular neighbourhood of *M* in *D* and

75 $$\begin{aligned} \pi :T \rightarrow M \text { a smooth map which restricts to the identity on} M \end{aligned}$$

If *D* is Riemannian $\pi$ can be chosen as in (41), but this is not necessary. Let $F \in \Gamma \text {Hom}(TN, TD)$ be a Stratonovich equation driven by an *N*-valued semimartingale *Z*, where *N* is another smooth manifold. We can then define the *M*-valued Stratonovich equation

76 $$\begin{aligned} M \times N \ni (y,z) \mapsto {\widetilde{F}}(y,z) {:}{=}T_y\pi \circ F(y,z) \in \text {Hom}(T_zN, T_yM) \end{aligned}$$

We call this Stratonovich SDE the *Stratonovich projection of* *F*.

Now consider the *Z*-driven, *D*-valued Schwartz-Meyer equation ${\mathbb {F}}\in \Gamma \text {Sch}(N,M)$. We can project this SDE to an SDE on *M* too, by

77 $$\begin{aligned} M \times N \ni (y,z) \mapsto {\widehat{{\mathbb {F}}}}(y,z) {:}{=}{\mathbb {T}}_y\pi \circ {\mathbb {F}}(y,z) \in \text {Sch}_{z,y}(N,M) \end{aligned}$$

We call this Schwartz-Meyer SDE the *Itô-jet projection of* ${\mathbb {F}}$.

If *N*, *D* and *M* all carry torsion-free connections we can interpret a section $F \in \Gamma \text {Hom}(TN, TD)$ as an Itô equation, and similarly for

78 $$\begin{aligned} M \times N \ni (y,z) \mapsto \overrightarrow{F}(y,z) {:}{=}T_y\pi \circ F(y,z) \in \text {Hom}(T_zN, T_yM) \end{aligned}$$

We call this Itô SDE the *Itô-vector projection* of *F*. Most often *D* will be Riemannian, so that Levi–Civita connections are defined on both *D* and *M*. Note that the Itô-vector projection is identical to the Stratonovich projection as a map, but the interpretations of the resulting sections as SDEs differ (and the Itô-vector projected SDE depends explicitly on the connections on all three manifolds). The names of these three projections are taken from [2], where they were first defined.

### Remark 5

(Naturality of the SDE projections) Assume we have a commutative square 
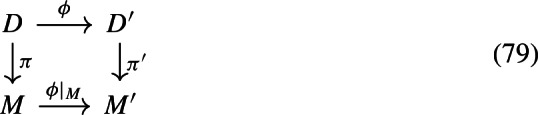
 where $\phi$ is a diffeomorphism, and $D,M,\pi$ are as above, and similarly for $D',M',\pi '$. Then functoriality of *T* and ${\mathbb {T}}$ imply that the Stratonovich and Itô-jet projections are natural in the sense that the squares 
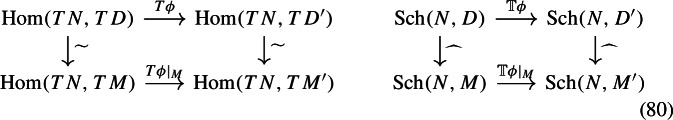


commute. The Itô-vector projection cannot be natural in the same way, since we are still free to modify the connections on all four manifolds. However, if $D,D'$ are Riemannian and $\phi$ is a global isometry, the corresponding statement does hold for the Itô-vector projection as well: this is by naturality of the Levi-Civita connection [13, Proposition 5.6].

### Remark 6

(The Itô-vector projection preserves local martingales) Although the Itô-vector projection is natural w.r.t. a smaller class of maps, it has the advantage of preserving the local martingale property: by this we mean that if the driver is a local martingale, so must the solution to the Itô-vector-projected SDE be. This is shown simply by the good behaviour of Itô equations w.r.t. manifold-valued local martingales.

### Remark 7

One might wonder whether it is possible to “push forward” SDEs according to an arbitrary smooth and surjective map $f :D \rightarrow D'$. If *f* is a surjective function admitting a smooth right inverse $\iota$, then we may write the pushforward of, say, the Stratonovich SDE $\text {d}X = F(X,Z) \circ \text {d}Z$ as $\text {d}Y = F(Z,\iota (Y)) \circ \text {d}Y$. This condition on *f* essentially corresponds to the condition (75). For general smooth surjective *f* (such as the bundle projection of a non-trivial principal bundle), however, we do not see a way of defining a new closed form SDE on $D'$.

We will now restrict our attention to the projections of ${\mathbb {R}}^d$-valued diffusions onto the embedded manifold *M*. Focusing on diffusions has the advantage of allowing us to use the maps (38) to compare the projections. In other words we can ask if the vertical rectangles in the diagram 
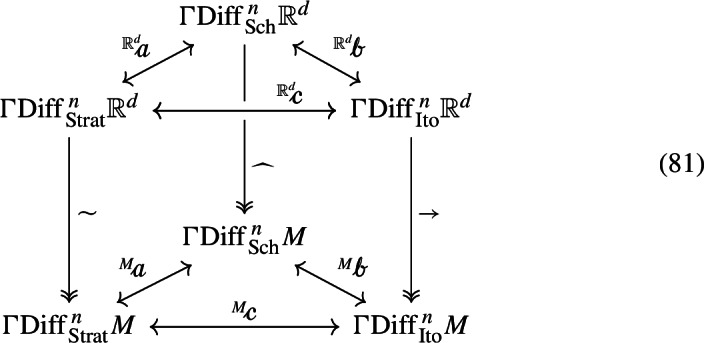
 commute (compare with (69), in which the equations on top already restrict to equations on *M*). We will show that they do not, and that all combinations of possibilities regarding their non-commutativity are possible. We recall the notation ${\overline{V}}_y {:}{=}P(y)V_y$,  and begin by considering the ${\mathbb {R}}^d$-valued Stratonovich SDE (54). By (44) the coefficients of the Stratonovich projection of this SDE will just be the projected coefficients: ${\widetilde{\sigma }}_\gamma = {\overline{\sigma }}_\gamma , {\widetilde{b}} = {\overline{b}}$, so that the resulting Stratonovich equation is

82 $$\begin{aligned} \begin{aligned} \text {d}Y_t&= {\overline{\sigma }}_\gamma (Y_t,t) \circ \text {d}W_t^\gamma + {\overline{b}}(Y_t,t) \text {d}t, \quad Y_0 = y_0 \in M \\&= \frac{\partial \pi }{\partial x^k}(Y_t) \sigma _\gamma ^k(Y_t,t) \circ \text {d}W^\gamma _t + \frac{\partial \pi }{\partial x^k}(Y_t) b^k(Y_t,t) \text {d}t \end{aligned} \end{aligned}$$

Throughout this paper we will use *X* for the initial SDE and *Y* to denote the projected SDE. Now assume we start with (56), and want an Itô SDE on *M*. We can still use the Stratonovich projection by converting the SDE to Stratonovich form as in (57), projecting as above, and converting back to Itô form (by (69) this last conversion can be seen to occur interchangeably in *M* or in ${\mathbb {R}}^d$). We have

83 $$\begin{aligned} \begin{aligned} \text {d}Y_t&= {\overline{\sigma }}_\gamma (Y_t,t) \circ \text {d}W^\gamma _t + P_k(Y_t) \bigg ( \mu ^k - \frac{1}{2} \sum _{\gamma = 1}^n \sigma ^h_\gamma \frac{\partial \sigma _\gamma ^k}{\partial x^h} \bigg )(Y_t,t) \text {d}t \\&= {\overline{\sigma }}_\gamma (Y_t,t) \text {d}W^\gamma _t + \bigg (\underbrace{{\overline{\mu }} + \frac{1}{2} \sum _{\gamma = 1}^n \bigg ({\overline{\sigma }}^l_\gamma \frac{\partial {\overline{\sigma }}_\gamma }{\partial x^l} - \sigma ^h_\gamma P_k\frac{\partial \sigma ^k_\gamma }{\partial x^h} \bigg )}_{{\widetilde{\mu }}} \bigg )(Y_t,t) \text {d}t \end{aligned} \end{aligned}$$

Using (53) we can split ${\widetilde{\mu }}$ in its orthogonal and tangential components: on *M* we have84with implied evaluation of all terms at (*y*, *t*).

We now move on to the Itô-jet projection. Let ${\mathbb {F}}\in \Gamma \text {Diff}_\text {Sch}^{\, n} {\mathbb {R}}^d$ as in (62), so that the Schwartz-Meyer equation it defines coincides with the Itô equation (56). We can then write (77) using matrix notation as

85 $$\begin{aligned} \begin{aligned} \begin{bmatrix} \text {d}Y_t \\ \frac{1}{2} \text {d}[Y]_t \end{bmatrix} = \begin{bmatrix} \frac{\partial \pi }{\partial x} &{} \frac{\partial ^2 \pi }{\partial x^2} \\ 0 &{} \frac{\partial \pi }{\partial x} \odot \frac{\partial \pi }{\partial x} \end{bmatrix}(Y_t)\begin{bmatrix} F &{} G \\ 0 &{} F \odot F \end{bmatrix}(Y_t,t) \begin{bmatrix} \text {d}W_t \\ \frac{1}{2} \text {d}[W]_t \end{bmatrix} \end{aligned} \end{aligned}$$

of which the first line reads

86 $$\begin{aligned} \begin{aligned} \text {d}Y_t&= \frac{\partial \pi }{\partial x^k}(Y_t) \bigg ( F^k_\gamma (Y_t,t)\text {d}W_t^\gamma + F^k_0(Y_t,t)\text {d}t + \frac{1}{2} \sum _{\gamma = 1}^n G_{\gamma \gamma }(Y_t,t) \text {d}t\bigg ) \\&\quad + \frac{1}{2} \sum _{ \gamma = 1}^n \frac{\partial ^2 \pi }{\partial x^i \partial x^j}(Y_t) F^i_\gamma F^j_\gamma (Y_t,t) \text {d}t \\&= {\overline{\sigma }}_\gamma (Y_t,t) \text {d}W^\gamma _t + \bigg ( \underbrace{{\overline{\mu }} + \frac{1}{2} \sum _{\gamma = 1}^n \frac{\partial ^2 \pi }{\partial x^i \partial x^j}\sigma ^i_\gamma \sigma ^j_\gamma }_{{\widehat{\mu }}} \bigg )(Y_t,t) \text {d}t \end{aligned} \end{aligned}$$

### Remark 8

We can write the Itô-jet-projected drift ${\widehat{\mu }}$ as the generator of the SDE, applied to the tubular neighbourhood projection $\pi$:

87 $$\begin{aligned} {\widehat{\mu }}(y,t) = \frac{\partial \pi }{\partial x^k} \mu ^k(t,y) + \frac{1}{2} \sum _{\gamma = 1}^n \frac{\partial ^2 \pi }{\partial x^i \partial x^j}\sigma ^i_\gamma \sigma ^j_\gamma (t,y) = ({\mathscr {L}}_t \pi )(y) \end{aligned}$$

In [1] the field of Schwartz morphisms ${\mathbb {F}}$ is taken to be induced by a (time-homogeneous) field of maps *f* as in (20). In this approach we can use functoriality of ${\mathbb {T}}$ to write

88 $$\begin{aligned} {\widehat{{\mathbb {F}}}}(y) = {\mathbb {T}}_y \pi \circ {\mathbb {F}}(y) = {\mathbb {T}}_y \pi \circ {\mathbb {T}}_0 f_{y} = {\mathbb {T}}_0 (\pi \circ f_y) \end{aligned}$$

thus obtaining an SDE defined by the field of (2-jets of) maps given by projecting the original field of maps onto *M* with the tubular neighbourhood projection $\pi$.

Finally, we consider the Itô-vector projection of (56). By (68), in coordinates this amounts to projecting (56) to the Itô SDE on *M* with diffusion coefficients given by ${\overline{\sigma }}_\gamma$ and drift

89 $$\begin{aligned} \overrightarrow{\mu }= \underbrace{{\overline{\mu }}}_{\in TM} + \underbrace{\frac{1}{2} \sum _{\gamma = 1}^n \frac{\partial ^2 \pi }{\partial x^i \partial x^j} {\overline{\sigma }}^i_\gamma {\overline{\sigma }}^j_\gamma }_{\in T^\bot M} \end{aligned}$$

To summarise, all three projections of the Itô equation (56) agree on how to map the diffusion coefficients, and the orthogonal components of the drift terms will all be fixed by the constraint (60), while their tangential projections are given by (respectively Stratonovich, Itô-jet, Itô-vector)90By calculations similar to (84) we can compute the projections of (54) in Stratonovich form: again, all three projections will orthogonally project the diffusion coefficients, and behave as follows on the Stratonovich drifts.91From now on we will consider (56) as being our starting point, unless otherwise mentioned, and thus refer to (90) when comparing the three projections.

We end this section with a brief comparison of the three projections. The three projections coincide if $\sigma _\gamma \in TM$ for $\gamma = 1,\ldots , n$ (which includes the ODE case $\sigma _\gamma = 0$), in which case the diffusion coefficients remain unaffected, and the tangent component of the projected drift is simply given by ${\overline{\mu }}$. If $\sigma _\gamma \in T^\bot M$ for $\gamma = 1,\ldots , n$ all three projections result in an ODE on *M*, and the Itô-jet and Itô-vector projections coincide. Another case in which the Itô-jet and Itô-vector projections coincide is when the second derivatives of $\pi$ vanish: this occurs in particular if *M* is embedded affinely, i.e. it coincides with some open set of an affine space of ${\mathbb {R}}^d$. All three projections forget the orthogonal part of the (Itô or Stratonovich) drift. We observe from (90) that the Itô-jet and Itô-vector projections of (56) only depend on the values of the Itô-coefficients on *M*. The Stratonovich projection, instead, could additionally depend on the tangential components of the derivatives of the diffusion coefficients in the direction of their normal components. Naturally, the situation is reversed when projecting (54): here it is the Stratonovich projection that only depends on the values of the coefficients on *M*, while the Itô-jet and -vector projections might depend on the mentioned derivative term.

### Example 5

(The projections in the case *M* time-dependent) Recalling Example 4 (and the map ${\widetilde{\pi }}$ defined therein) we may ask whether there is a way to consider the three SDE projections in the case of *M* time-dependent. The most natural way to define this is to consider, as done in (71), the joint equation satisfied by $(t,X_t)$, project its coefficients in the three ways onto ${\widetilde{M}}$, thus obtaining a solution of the form $(t,Y_t)$: this uses that ${\widetilde{\pi }}^0(t,y) = t$ (with time the $0^\text {th}$ coordinate), which is instead not necessarily satisfied by the Riemannian tubular neighbourhood projection onto ${\widetilde{M}}$. It is easily checked that the formulae (90) for the tangential component of the drift of $Y_t$ continue to hold with the substitution of $\pi _t$ for $\pi$ (so that also the projection onto the tangent space *P* is now time-dependent), whereas in all three cases the orthogonal component of the drift picks up the term ${\dot{\pi }}_t$, needed to keep the process on the evolving manifold $M_t$. In particular, in the Itô-jet case we have

92 $$\begin{aligned} {\widehat{\mu }}(y,t) = ({\mathscr {L}}_t \pi _t)(y) + {\dot{\pi }}_t(y) = \widetilde{{\mathscr {L}}} {\widetilde{\pi }} (t,y) \end{aligned}$$

where ${\mathscr {L}}_t$ is the generator of *X* and $\widetilde{{\mathscr {L}}}$ is that of $(t,X_t)$ (which can be considered as being a time-homogeneous Markov process). This identity extends the observation made in Remark 8. The same term ${\dot{\pi }}_t$ should be added to the Stratonovich drifts (91) for the extension to the case of *M* time-dependent.

## The optimal projection

In the previous section we showed how to abstractly project manifold-valued SDEs onto submanifolds in three (possibly) different ways, and specialised these constructions to the case of $M \hookrightarrow {\mathbb {R}}^d$-valued diffusions. In this section we will seek the *optimal* projection of an SDE for $X_t$, which we write in Itô form as (56). Let93be the *M*-valued SDE to be defined, which we write in ${\mathbb {R}}^d$-coordinates. Its coefficients  and  are to be treated as unknowns, to be determined by the optimisation criteria that involve the minimisation of the quantities

94 $$\begin{aligned} E[|Y_t - X_t|^2], \qquad E[|Y_t - \pi (X_t)|^2], \qquad |E[Y_t - X_t]|^2 \end{aligned}$$

asymptotically for small *t*. Before we define the optimality criteria precisely, it is important to note that such expectations are undefined if the solution to either SDE is explosive, or, in the second case, even if it exits the tubular neighbourhood of *M* on which $\pi$ is defined. The problem must be slightly changed so as to ensure that we are minimising a well-defined quantity. One option is to take the above expectations on the event $\{t \le \tau _r\}$, where

95 $$\begin{aligned} \tau _r {:}{=}\min \{t \ge 0 : |(X_t,Y_t) - (y_0,y_0)|^2 \ge r^2 \} \end{aligned}$$

for some suitable $r>0$. However, since for such optimality criteria the values of the vector fields of both SDEs outside the ball $B_{(y_0,y_0)}(r) \subseteq {\mathbb {R}}^{2d}$ are irrelevant, it is simpler to just assume that they vanish outside $B_{(y_0,y_0)}(2r)$. Since the optimisation criteria will only determine the value of ,  at the initial condition, this is really only an assumption on $\sigma$ and $\mu$. The following proposition reassures us that, at least in well-behaved cases, this does not alter the problem in a way that interferes with the optimisation (which, as will be seen shortly, only involves the Taylor expansions of order 2 of (94) in $t = 0$).

### Lemma 1

Let $X,Y,y_0,\tau _r$ be as above, *U* a neighbourhood of $(y_0,y_0)$ in ${\mathbb {R}}^{2d}$ and assume that there exists deterministic $\varepsilon >0$ s.t. $X_t,Y_t \in U$ for $t \in [0,\varepsilon ]$. Let $f :U \times [0,\varepsilon ] \rightarrow {\mathbb {R}}$ be continuous s.t. $f(y_0,y_0,0) = 0$, and assume moreover that $E[\max _{0 \le t \le \varepsilon }|f(X_t,Y_t,t)|] < \infty$ (this holds, in particular, under the global Lipschitz assumptions that guarantee SDE exactness [15, Theorem 11.2]). Then for any $r > 0$ with ${\overline{B}}_r(y_0,y_0) \subseteq U$

96 $$\begin{aligned} E[f(X_t,Y_t,t)] - E[f(X_t,Y_t,t);t<\tau _r] \end{aligned}$$

belongs to $O(t^n)$ for all $n \in {\mathbb {N}}$ as $t \rightarrow 0$.

### Proof

Fix *r*, and let $\tau {:}{=}\tau _r$. The Itô formula yields the decomposition $|(X_t,Y_t) - (y_0,y_0)|^2 = L_t + A_t$ with $L_t$ sum of Brownian integrals and $A_t$ time integral, all of which for $t \le \tau \wedge \varepsilon$ have bounded integrand (by continuity of the SDE coefficients and compactness of ${\overline{B}}_r(y_0,y_0) \times [0,\varepsilon ]$). $[L]_t$ can be expressed as a time integral with bounded integrand: let $R>0$ bound the sum of the absolute values of all integrands mentioned for $t \in [0,\tau \wedge \varepsilon ]$. Then, still for $t \le \tau \wedge \varepsilon$ we have $|A_t|, [L]_t \le Rt$, and for any $\xi > 0$ it holds that $|(X_t,Y_t) - (y_0,y_0)|^2 \le L_t + R\xi$ for $0 \le t \le \varepsilon \wedge \xi$. Letting $\xi {:}{=}r^2/(3R)$, on $[0,\varepsilon \wedge \xi ]$ we have

97 $$\begin{aligned} \begin{aligned} P[t \ge \tau ]&= P\big [\max _{0 \le s \le t}|(X_s,Y_s) - (y_0,y_0)|^2 \ge r^2 \big ] \\&= P\big [\max _{0 \le s \le \tau \wedge t}|(X_s,Y_s) - (y_0,y_0)|^2 \ge r^2 \big ] \\&\le P\big [\max _{0 \le s \le \tau \wedge t} L_s> r^2/2 \big ] \\&= P\big [\max _{0 \le s \le t} L_{\tau \wedge s} > r^2/2 \big ] \\&\le \exp \bigg (-\frac{r^4}{4Rt} \bigg ) \end{aligned} \end{aligned}$$

by the tail estimate [15, Theorem 37.8 p.77]. Now, for $t \in [0,\varepsilon \wedge \xi ]$ by Cauchy-Schwarz

98 $$\begin{aligned} \begin{aligned}&\big | E[f(X_t,Y_t,t)] - E[f(X_t,Y_t,t);t<\tau ] \big | \\&\quad = \big |E[f(X_t,Y_t,t);t\ge \tau ]\big | \\&\quad \le E[f(X_t,Y_t,t)^2]^{1/2} P[t \ge \tau ]^{1/2} \\&\quad \le E\Big [\max _{[0,t]}f(X_s,Y_s,s)^2\Big ]^{1/2} P[t \ge \tau ]^{1/2} \\&\quad \lesssim \exp \bigg (-\frac{r^4}{4Rt} \bigg ) \end{aligned} \end{aligned}$$

since the first factor also vanishes as $t \rightarrow 0$, by the hypotheses on *f*, *X*, *Y* and dominated convergence. $\square$

We proceed with the constrained optimisation problem, assuming all SDE coefficients to be compactly supported; this means all local martingales involved will be martingales, and that we may use Fubini to pass to the expectation inside integrals in $\text {d}t$. If we can write the Taylor expansion of the strong error

99 $$\begin{aligned} E \big [ |Y_t - X_t|^2\big ] = a_1 t + a_2 t^2 + o(t^2) \end{aligned}$$

a first goal could be to minimise the leading coefficient $a_1$ (of course there is no constant term because $Y_0 = y_0 = X_0$). Using Itô’s formula, and intending with $\simeq$ equality of differentials up to differentials of martingales, we haveWe now compute the expectation:and differentiating, with reference to (99) we have100where $\frac{\text {d}}{\text {d}t} \big |_0^+$ denotes differentiating from the right. Since $a_1$ only depends on the diffusion coefficients, its minimisation is expressed by the constrained optimisation problem whose solution is simply given by projecting the $\sigma _\gamma$’s onto *TM*:101Here we have omitted evaluation at the initial condition $(0,y_0)$. Since we have not obtained a condition on  our SDE (93) is still underdetermined, and the condition would be satisfied by the Stratonovich projection of (56).

One idea to obtain a condition on  would be to minimise $a_2$ in (99). This attempt, however, has the drawback that we are minimising the second Taylor coefficient of a function without its first vanishing (unless the $\sigma _\gamma$’s are already tangent to start with: in this case the minimisation of $a_2$ can be seen to result in the three projections, which all coincide). Although this approach is discussed in [2], we will not do so here, as there are more sound optimisation criteria. Indeed, we can look at the Taylor expansion of the weak error

102 $$\begin{aligned} |E[Y_t - X_t ]|^2 = b_2 t^2 + o(t^2) \quad \text {as } t \rightarrow 0^+ \end{aligned}$$

We compute the term on the left as103and104which confirms that (102) lacks a linear term, and we have105Requiring the minimisation of $b_2$ is thus independent of the minimisation of $a_1$ above, and results in the constrained optimisation problem106A quick glance at (90) shows we have proven the following

### Theorem 1

(Optimality of the Itô-vector projection) The coefficients  of the *M*-valued SDE (93) that solve the constrained optimisation problem

107 $$\begin{aligned} {\left\{ \begin{array}{ll} minimise \, a_1\, in\, (99)\, and\, b_2\, in\, (102) \\ subject\, to\, (60) \end{array}\right. } \end{aligned}$$

for all initial conditions $X_0 = Y_0 = y_0 \in M$ are given (uniquely for $t=0$) by the Itô-vector projection of the original SDE (56).

### Remark 9

In defining the three projections in Sect. 4 we intended for the projected coefficients to still be time-dependent if the original ones were. The optimality requirement only fixes the coefficients at the initial condition, at time 0, i.e. . To retain the time-dependence we may consider the optimisation involving all time-translated initial conditions $Y_{t_0} = y_0$.

### Remark 10

Note that the form (Itô or Stratonovich) the initial SDE is provided in is irrelevant: if we had begun with (54) instead of (56) the optimality criterion would still have led us to the Itô-vector projection, which for the Stratonovich drift would have taken the form $\overrightarrow{b}$ in (91). The only reason to start with an Itô SDE is that the calculations are simpler, and it is possible to express the optimal coefficients as functions of the values of the coefficients of the original SDE, without reference to their derivatives.

The optimisation of 1 has the disadvantage of coming from the two separate minimisations of $a_1$ and $b_2$, which are Taylor coefficients of different quantities. There is a different way of arriving at coefficients by successively minimising the Taylor coefficients of the same quantity, with the first minimisation resulting in a null term. The idea is to consider

108 $$\begin{aligned} E\big [|Y_t - \pi (X_t)|^2 \big ] = c_1 t + c_2 t^2 + o(t^2) \end{aligned}$$

where *X*, *Y* are respectively as in (56) and (93), again assuming the coefficients of *Y* vanish outside a small neighbourhood contained in the domain of $\pi$ so as to make the expectation well-defined. The map $\pi$ is the one defined in (41), although it can more generally satisfy (75). Letting  resume their status as unknowns, we proceed with the calculations.and109and therefore110(evaluation at $(y_0,0)$ is implied). Thus $c_1$ vanishes if and only if . Continuing as before we have111for some smooth function *f* (*J* denotes Jacobian and *H* Hessian), which we denote $f_t$ for short; the differentials $\text {d}(\ldots )$ can be ignored, since their factors will vanish when evaluated below.112The constrained optimisation problem for the minimisation of $c_2$ conditional on the previous minimisation of $c_1$ is thus given by113Comparing with (87) we see that we have proven the following

### Theorem 2

(Optimality of the Itô-jet projection) The coefficients  of the *M*-valued SDE (93) that solve the constrained optimisation problem

114 $$\begin{aligned} {\left\{ \begin{array}{ll} \textit{minimise}\, c_1\, and\, c_2,\, \textit{conditionally}\, \textit{on}\, \textit{the}\, \textit{minimisation}\, \textit{of}\, c_1,\, \textit{in}\, (108) \\ \textit{subject to } (60) \end{array}\right. } \end{aligned}$$

for all initial conditions $X_0 = Y_0 = y_0 \in M$ are given (uniquely for $t=0$) by the Itô-jet projection of the original SDE (56).

Remarks analogous to Remarks 9 and 10 hold for 2. The Itô-vector and Itô-jet projection therefore satisfy different optimality properties, while the Stratonovich projection is suboptimal in both senses. We end the section with the extension of the optimisations to the case of *M* time-dependent.

### Example 6

(Optimality for *M* time-dependent) Recall the case in which the submanifold *M* depends smoothly on time, for which we can define similar versions of all three projections (Example 5). For 1 the optimisation criterion does not require reformulation, while the constraint is modified as described in Example 4: therefore the Itô-vector projection remains optimal in the case of *M* time-dependent. For 1 the natural generalisation is given by substituting $\pi _t$ for $\pi$ in (108). Since $|y - \pi _t(x)| = |(t,y) - {\widetilde{\pi }}(t,x)|$, by the definition of the Itô-jet projection in the case of *M* time-dependent (and since the calculations in this section never relied on $\pi$ being the Riemannian tubular neighbourhood projection), we have that the time-dependent Itô-jet projection (92) is optimal in this case too.
